# Transcriptomic and metabolomic profiling of *Zymomonas mobilis *during aerobic and anaerobic fermentations

**DOI:** 10.1186/1471-2164-10-34

**Published:** 2009-01-20

**Authors:** Shihui Yang, Timothy J Tschaplinski, Nancy L Engle, Sue L Carroll, Stanton L Martin, Brian H Davison, Anthony V Palumbo, Miguel Rodriguez, Steven D Brown

**Affiliations:** 1 Biosciences Division and BioEnergy Science Center, Oak Ridge National Laboratory, Oak Ridge, TN 37831, USA; 2 North Carolina State University, 840 Main Campus Drive, Raleigh, NC 27606, USA

## Abstract

**Background:**

*Zymomonas mobilis *ZM4 (ZM4) produces near theoretical yields of ethanol with high specific productivity and recombinant strains are able to ferment both C-5 and C-6 sugars. *Z. mobilis *performs best under anaerobic conditions, but is an aerotolerant organism. However, the genetic and physiological basis of ZM4's response to various stresses is understood poorly.

**Results:**

In this study, transcriptomic and metabolomic profiles for ZM4 aerobic and anaerobic fermentations were elucidated by microarray analysis and by high-performance liquid chromatography (HPLC), gas chromatography (GC) and gas chromatography-mass spectrometry (GC-MS) analyses. In the absence of oxygen, ZM4 consumed glucose more rapidly, had a higher growth rate, and ethanol was the major end-product. Greater amounts of other end-products such as acetate, lactate, and acetoin were detected under aerobic conditions and at 26 h there was only 1.7% of the amount of ethanol present aerobically as there was anaerobically. In the early exponential growth phase, significant differences in gene expression were not observed between aerobic and anaerobic conditions via microarray analysis. HPLC and GC analyses revealed minor differences in extracellular metabolite profiles at the corresponding early exponential phase time point.

Differences in extracellular metabolite profiles between conditions became greater as the fermentations progressed. GC-MS analysis of stationary phase intracellular metabolites indicated that ZM4 contained lower levels of amino acids such as alanine, valine and lysine, and other metabolites like lactate, ribitol, and 4-hydroxybutanoate under anaerobic conditions relative to aerobic conditions. Stationary phase microarray analysis revealed that 166 genes were significantly differentially expressed by more than two-fold. Transcripts for Entner-Doudoroff (ED) pathway genes (*glk, zwf, pgl, pgk, and eno*) and gene *pdc*, encoding a key enzyme leading to ethanol production, were at least 30-fold more abundant under anaerobic conditions in the stationary phase based on quantitative-PCR results. We also identified differentially expressed ZM4 genes predicted by The Institute for Genomic Research (TIGR) that were not predicted in the primary annotation.

**Conclusion:**

High oxygen concentrations present during *Z. mobilis *fermentations negatively influence fermentation performance. The maximum specific growth rates were not dramatically different between aerobic and anaerobic conditions, yet oxygen did affect the physiology of the cells leading to the buildup of metabolic byproducts that ultimately led to greater differences in transcriptomic profiles in stationary phase.

## Background

Recent high oil prices, concerns over energy security, and environmental goals have reawakened interest in producing alternative fuels via large-scale industrial fermentations. The potential and challenges involved in supplanting a substantial amount of petroleum derived transportation fuels with fuels derived from renewable resources such as ethanol from lignocellulosic materials has been the focus of several studies and reviews [1-4]. The development and deployment of ethanologenic microorganisms will be one critical component in the successful production of fuel ethanol in industrial-scale quantities.

Essential traits for an industrial microorganism include high ethanol yield, tolerance, and productivity (> 90% of theoretical, > 40 g L^-1^, > 1 g L^-1 ^h^-1^, respectively); robust growth with simple, inexpensive growth requirements in conditions that retard contaminants (eg higher temperatures); and inhibitor tolerance, as reviewed previously [5]. Higher tolerance, productivity values and other positive industrial attributes have been reported for *Z. mobilis*, as reviewed previously [6]. Ethanol tolerance comparable up to 85 g L^-1^(11% v/v) have been reported for *Z. mobilis *continuous culture and up to 127 g L^-1 ^(16% v/v) in batch culture and productivities of 120–200 gL^-1 ^h^-1 ^in continuous processes with cell recycle [6]. *Saccharomyces *yeasts have been the preferred industrial biocatalyst for fuel ethanol production, although genetically engineered bacterial species such as Gram-negative bacteria *Escherichia coli, Zymomonas mobilis*, and *Klebsiella oxytoca *as well as Gram-positive bacteria *Bacillus subtilis *and *Corynebacterium glutamicum *are in development to address commercially important inoculum requirements [5,7,8]. Indeed, a newly formed partnership between the DuPont and Broin companies will utilize recombinant strains of *Z. mobilis *for bio-ethanol fermentation from the lignocellulosic residues such as corn stover [9].

*Z. mobilis *ferments glucose, fructose, and sucrose producing ethanol and carbon dioxide via the Entner-Doudoroff (ED) pathway, utilizing pyruvate decarboxylase and alcohol dehydrogenase enzymes (see [6,10-12] for reviews). *Z. mobilis *is not a classic facultative organism, rather it is aerotolerant, negating oxygen requirements in fermentations and the need for expensive oxygen transfer. The unusual physiology of *Z. mobilis *generates only one mole of ATP per mole of glucose, which results in low biomass production and greater carbon being available for fermentation products under anaerobic conditions. Its desirable ethanologenic attributes also include: high sugar uptake rates, near theoretical ethanol yields, high ethanol tolerance and generally regarded as safe (GRAS) status. Wild-type *Z. mobilis *can only utilize a limited range of substrates; however, it is amenable to genetic manipulation and recombinant strains have been developed to ferment pentose sugars such as xylose and arabinose [13-15]. Seo et al (2005) reported the first genome sequence for *Z. mobilis *ZM4 [16] and the U. S. Department of Energy's Joint Genome Institute (JGI)  has announced plans to sequence the genomes of additional *Z. mobilis *strains in the near future. The ZM4 genome sequence provides new opportunities for fundamental insight into the physiology and gene function and regulation of this unique microorganism and likely improvements in strain development [17]. The presence of oxygen during *Z. mobilis *fermentations has been observed to negatively affect cell and ethanol yields with acetic acid and acetaldehyde accumulating in the medium [12,18-20]. However, there are also reports that respiration inhibition by cyanide stimulates *Z. mobilis *growth aerobically [21]. Many aspects of end-product inhibition and basic aspects of *Z. mobilis *physiology remain unexplored [11].

In this study, we combined transcriptomic profiling with metabolomic measurements of aerobic and anaerobic *Z. mobilis *fermentations to elucidate the molecular mechanisms of ZM4s growth with and without oxygen. Metabolomics is the study of the low molecular weight metabolites present in and around a biological organism at a given time [22,23]. To date, there are few reports of bacterial mRNA expression-profiling using whole genome microarray analysis conducted in conjunction with metabolomics [24]. We confirmed maintenance of anaerobiosis was important for maximizing ethanol yields since acetaldehyde, acetate, lactate and acetoin accumulated and ethanol was present in lower concentrations under controlled aerobic conditions. Our data revealed alterations in mRNA expression profiles, differences in intra- and extracellular metabolites, as well as identification of differential expression for coding sequences predicted by TIGR  and not predicted in the primary annotation. These data provide global insight into potential molecular mechanisms of *Z. mobilis *aerobic and end-product stress responses.

## Results

### ZM4 grows and consumes glucose faster under anaerobic conditions

**Figure 1:**
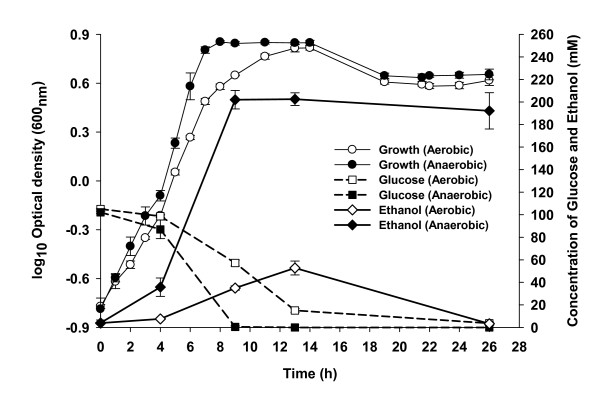
***Z. mobilis *fermentations under anaerobic and aerobic conditions**. Mean values for triplicate fermentors are shown for each condition ± standard deviation (bars).

The presence of oxygen negatively affected glucose consumption and growth in *Z. mobilis *ZM4 fermentations (Fig. 1). Anaerobic fermentation led to a maximal culture density of 7.0 OD_600 _units approximately 9 h post-inoculation, while *Z. mobilis *did not reach its highest culture density of 6.5 OD_600 _units until 13 h post-inoculation under aerobic conditions despite initial inocula concentrations being slightly greater than the former condition. *Z. mobilis *also consumed glucose more slowly under aerobic conditions, with more than half of the initial aerobic glucose concentration (105 mM) remaining 9 h post-inoculation. Under anaerobic conditions 99.5% of the glucose had been utilized at this time point. When *Z. mobilis *growth reached its peak after 13 h under aerobic conditions 14% of the glucose remained with the remainder consumed without cell growth (Fig. 1). Despite these differences and differences in extracellular metabolite production (below), the maximum specific growth rates were not dramatically different, which were estimated to be 0.45 h^-1 ^and 0.55 h^-1 ^between aerobic and anaerobic conditions, respectively. Fermentor pH, dissolved O_2 _tension (DOT), and agitation speed were well-controlled, which was indicated by the mean fermentor pH, DOT, and agitation values for each condition (see Additional file 1), and DOT trend data for each condition (see Additional file 2).

### *Z. mobilis *extracellular metabolite production

**Figure 2:**
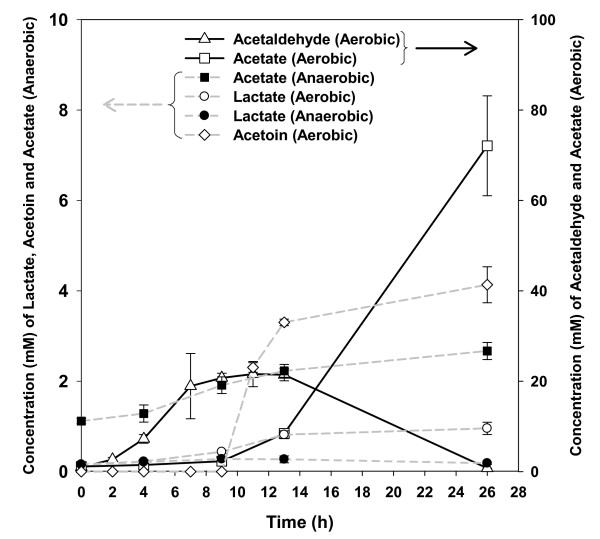
***Z. mobilis *extracellular fermentation product analysis**. The mean value for each metabolite identified by GC analysis from three independent fermentors for each condition is presented ± standard deviation (bars).

Previous reports have indicated that ethanol production was decreased and other end-products such as acetaldehyde, acetate and acetoin were increased under aerobic conditions [12,18-20,25]. GC and HPLC were used to quantify and compare the kinetics of ethanol, acetate, acetaldehyde, lactate and acetoin production during aerobic and anaerobic fermentation processes and extracellular metabolites were often measured by more than one approach, which confirmed the observed trends. The more rapid production of ethanol under anaerobic conditions also corresponded with increased glucose uptake and growth under these conditions (Fig. 1). The ethanol concentration remained relatively stable post-peak production in anaerobic fermentations. In contrast, at 13 h the ethanol concentration dropped sharply from 52.7 mM to 3.2 mM at the end of the 26 h fermentation during aerobic fermentation. The decrease in ethanol concentration during this time was matched in nearly stoichiometric increases of acetate production, which went from 8.4 mM at 13 h to 72.1 mM at 26 h (Fig. 2). GC data also showed several other minor unidentified metabolites were being produced during aerobic fermentations relative to anaerobic conditions (data not shown). The concentration of acetaldehyde increased during the exponential growth and dropped appreciably during stationary phase, while acetoin was detected during stationary phase (Fig. 2). Lactate trended similarly to ethanol and acetoin profiles for respective conditions. The anaerobic ethanol yield at 13 h was 0.497 g/g of glucose or 97% theoretical and less than 1% of the yield went to solvent products other than ethanol or CO_2 _(Fig. 1). In contrast, under aerobic conditions at 13 h 0.14 g/g of glucose or 27% theoretical yield was obtained for ethanol and by 26 h around 3.2 mM ethanol remained (Fig. 1). The total measured solvents produced aerobically was 101 g/L, which was approximately 50% of theoretical total solvent yield at 13 h and at 26 h these figures had dropped to 76 g/L or approximately 37% of theoretical total solvent yield. As cell biomass (as measured by optical density) was approximately equivalent under the two conditions more carbon went to maintenance energy under aerobic conditions.

### *Z. mobilis *intracellular metabolomic profiles

The physiological status of ZM4 was investigated further by GC-MS analysis of stationary phase intracellular metabolomic profiles for each respective condition. GC-MS identified a large number of metabolites, however, we present those that showed differences in relative abundance and showed some statistical significance. We observed differences in relative abundance profiles for metabolites related to central carbon metabolism, although only several were considered significantly different during the stationary phase time point we examined. We present a relative comparison of the metabolite difference from two conditions in keeping with current practices, while recognizing accessing metabolites like ATP will require modified procedures [22]. Metabolite identification and analysis gave 20 metabolites that were different between anaerobic and aerobic cultures, with five out of the 20 less abundant in anaerobic *Z. mobilis*. Twelve metabolites were considered significantly different with a *p*-value less than 0.1 (Table 1). The inclusion of a greater number of replicates would have increased analysis power and possibly increased the number of metabolites considered significantly different between anaerobic cells and those exposed to oxygen.

**Table 1:** Intracellular metabolomic profile of ZM4 during anaerobic and aerobic fermentation at 26 h.

**Metabolite**	**Ratio (Anaerobic/Aerobic)**	***p*-value**
Glucose 6-P	2.7	0.089
Mannose 6-P	3.2	0.009
Glycerate	0.65	0.10
Glucose	0.16	0.14
Gluconate	0.17	0.33
2-Phosphoglycerate	3.8	0.053

Alanine	0.33	0.015
Valine	0.32	0.031
Lysine	0.35	0.097
Isoleucine	0.32	0.17
Leucine	0.29	0.13
Phenylalanine	0.31	0.23
Serine	0.17	0.19
Threonine	0.27	0.21

lactate	0.50	0.01

4-hydroxybutanoate	7.60	0.029
Ribitol	0.06	0.034
Trehalose	0.34	0.13
Unknown 285 18.19	3.0	0.074
Unknown 348 11.22 AA	0.72	0.024

Glucose-6-phosphate and 2-phosphoglycerate were 2.7 and 3.8 fold more abundant under anaerobic conditions (*p *= 0.089 and 0.053, respectively), while others in the ED pathway may have been at greater levels under aerobic conditions. The ability to detect labile phosphorylated intermediates such as these, in the absence of accumulation phosphate was indicative of adequate sample preparation for GC-MS analysis of the metabolites analyzed in the present study. Aerobic *Z. mobilis *contained more of the glucose substrate, as well as glycerate, and gluconate as compared to the anaerobic cells, while other intermediate sugar metabolites that included glucose-6-phosphate, 2-phosphoglycerate and mannose-6-phosphate were more abundant within the anaerobic fermenting *Z. mobilis *(Table 1). The transcriptomic profiles of Entner-Dondoroff and pyruvate metabolic pathways showed higher expression values for these genes under anaerobic conditions even though extracellular glucose had been consumed by 26 h (Fig. 1, see Additional file 3). All of the differentially detected amino acids were at lower concentrations within anaerobic fermenting *Z. mobilis *(Table 1). The other metabolites showing differences included lactate, 4-hydroxybutanoate, ribitol, trehalose and unknown metabolites. Trehalose was 2.9-fold more abundant within aerobic *Z. mobilis *compared to anaerobic cultures, and 4-hydroxybutanoate was the most abundant metabolite detected within anaerobic condition at 7.6-fold higher levels compared to aerobic conditions (Table 1).

### Transcriptome comparison of *Z. mobilis *aerobic and anaerobic fermentations

The global transcriptional response of *Z. mobilis *ZM4 to aerobic stress was examined in a time series DNA microarray experiment for early exponential phase (3 h post-inoculation) and stationary phase (26 h post-inoculation) under aerobic and anaerobic conditions with whole-genome microarrays. In this study, we have presented experimental data of 166 differentially expressed genes at statistically significant values (-log_10_(*p*) = 5.43) with a number of 11 putative protein-coding sequences in strain ZM4 (ATCC31821) that were not originally described in the primary genome annotation (see Additional file 4, 5). We have deposited the entire microarray dataset including exponential and stationary phase at Gene Expression Omnibus (GEO, ) database with the accession number of GSE10302 so interested parties can conduct their analyses.

**Figure 3:**
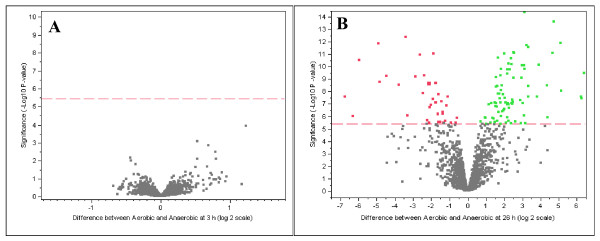
**Volcano plot result from JMP Genomics analysis showing significantly differentially expressed genes at 3 h (A) and 26 h (B) post-inoculation at 30°C**. Green dots indicate oxygen up-regulated genes and red dots indicate the oxygen down-regulated genes. Grey colored dots were not considered significantly differentially expressed. The X axis shows the difference values between aerobic and anaerobic fermentations based on a log_2 _scale. The Y axis shows statistical significance values for expression values, based on a -log_10 _*p*-value. The red dashed line shows the statistical significance cut-off used in this study.

**Figure 4:**
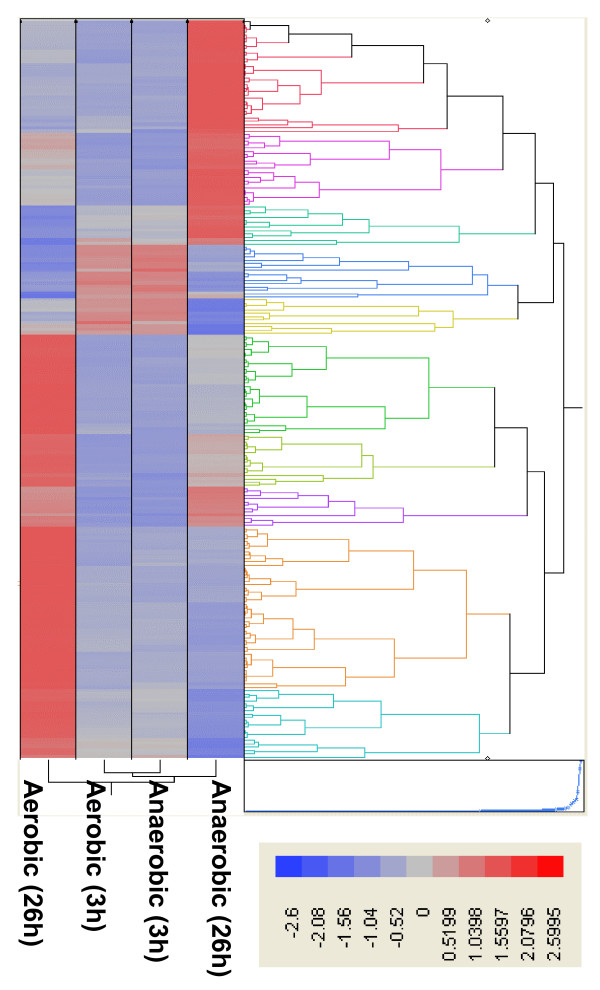
**Hierarchical cluster analysis of significantly differentially expressed ZM4 genes for aerobic and anaerobic fermentations at 3 and 26 h**. Gene expression values were clustered based on their log_2 _based expression values using JMP Genomics 3.0. Negative numbers (colored blue) indicates less relative gene expression aerobically, and positive numbers (colored red) indicate greater relative gene expression anaerobically.

Gene expression during the early exponential phase was surprisingly similar between anaerobic and aerobic conditions used in the present study (Fig. 3A). Eight genes were discovered that were considered differentially expressed at a significant level utilizing the ANOVA model using the False Discovery Rate (data not shown). Of these, only one gene (ZMO1752, encoding a hypothetical protein) showed a greater than a two-fold difference in relative expression levels. To confirm the microarray results, seventeen genes involving in ED and pyruvate pathways and different cellular functions were chosen for the qPCR analysis (see Additional file 6, 7). The data showed that qPCR was more sensitive and showed greater fold change differences compared to the microarray analysis, which was in keeping with previous reports [26]. We describe here only a rigorous analysis of stationary phase genes in this study and allow interested parties to conduct their own analyses on the entire dataset, which is available publicly through the GEO database. In the stationary phase 166 genes were significantly differentially expressed between anaerobic and aerobic conditions (Fig. 3B, 4, see Additional file 4, 5). This time point also showed the largest differences in extracellular metabolite profiles (Fig. 2). Fifty-five genes were up-regulated at 26 h post-inoculation under aerobic conditions and 111 genes were down-regulated (see Additional file 4, 5). Approximately two thirds of the genes down-regulated in the presence of oxygen for this time point were related to metabolism (see Additional file 4). In the presence of oxygen, genes related to regulation, cell processes, transport, and unknown function showed greater expression as compared to anaerobic conditions. Nearly half of the genes showing greater expression aerobically remain uncharacterized (see Additional file 5).

In the stationary phase, ED pathway mRNAs such as *glk, zwf, pgl, pgk*, and *eno *as well as ethanol fermentation gene transcripts like *pdc *and *adhB *were shown to be more abundant (at least two-fold) under anaerobic conditions by microarray analysis (see Additional file 3, 4, 7). In this study we observed that one arginyl-tRNA synthetase involved in ribosome-mediated polypeptide synthesis, eight genes related to ribosomal protein synthesis and amino acid and co-factor biosynthetic genes such as *leuC, trpB, argC, ilvI, ilvC, thrC, thiC*, and *ribC *showed greater expression under anaerobic conditions (see Additional file 4). Metabolomics data showed that a number of detected amino acids were generally less abundant in anaerobic fermenting cells compared to aerobic cells (Table 1).

Our microarray data identified a cysteine desulfurase (ZMO1022), thiosulfate sulfurtransferase (ZMO1460) and a [2Fe-2S] binding domain family protein (NT01ZM1467) as being up-regulated under aerobic conditions (see Additional file 5) and suggested the metabolism of sulfur compounds was impacted by aerobic conditions. However, we did not observe any trends related to sulfur-containing metabolites in our metabolomics dataset. Genes related to sensing and responding to environmental signals including three chemotaxis genes *cheX *(ZMO0084), *motD *(ZMO0641) and *fliD *(ZMO0651), six transcriptional regulators including a two-component signal transduction (TCSTS) histidine kinase (ZMO1216) and response regulator (ZMO1387) were up-regulated during aerobic fermentation (see Additional file 5). We also observed that several ATP synthase subunit genes (alpha and beta) were expressed less under aerobic conditions. The expression of NAD synthetase gene (*nadE*), involved in nicotinamide adenine dinucleotide *de novo *biosynthesis and salvage pathways was approximately 9-fold greater in the presence of oxygen (see Additional file 5). Several flavoprotein transcripts, nitroreductase (*tdsD*) and flavodoxin (*nifF*), were approximately 9-fold more abundant under aerobic conditions.

Expression of a number of stress response genes was found to be greater in the presence of oxygen or by-products in the stationary phase. Under aerobic conditions ZMO1097 encoding a thioredoxin was induced approximately four-fold, ZMO1830 (*fdxB*) encoding a ferredoxin showed six-fold induction, ZMO1732 (*ahpC*) encoding an alkyl hydroperoxide reductase showed 18-fold greater expression, ZMO0279 encoding a cold-shock protein was induced two-fold, and a glutathione S-transferase family protein encoded by ZMO1118 was induced eight-fold. The *E. coli *alternative sigma factor *rpoH *plays an important role in overcoming oxidative stress responses [27] and *rpoH *(ZMO0749) was induced approximately 32-fold under aerobic conditions via q-PCR in our study (see Additional file 7). Other regulators with greater expression levels under aerobic condition include ZMO1121 encoding a MerR family regulator, ZMO1216 encoding a two-component signal transduction histidine kinase, ZMO1387 encoding a two-component response regulator, and ZMO1063 (*pspA*) encoding a sigma 54-dependent transcription suppressor (see Additional file 5). A number of these microarray expression values were confirmed by qPCR (see Additional file 6, 7).

The microarray data also indicated that a number of CDS predicted by TIGR but not present in the primary annotation [16] were differentially expressed between the two conditions. Transcripts for NT01ZM1467 encoding a Fe-S binding domain family protein and nine other ORFs with unknown functions were more abundant under aerobic conditions representing approximately 9% of all the genes up-regulated under these conditions (see Additional file 5). The only example of an up-regulated gene (~six-fold) without primary locus identification during anaerobic fermentation was NT01ZM0869, which encodes a putative arginyl-tRNA synthetase (see Additional file 4).

## Discussion

*Z. mobilis *has a number of positive attributes as an ethanogen and is a leading current generation candidate microorganism for use in commercial scale fermentations to produce fuel ethanol [6,10]. In the present study, we confirmed oxygen levels were important in *Z. mobilis *growth rates, ethanol production and altered metabolite pools (Fig. 1, 2, Table 1). Anaerobic *Z. mobilis *fermentations utilized glucose more rapidly and grew more quickly with concomitant increases in ethanol productivity and yield as compared to aerobic *Z. mobilis *cultures (Fig. 1). Both intra- and extracellular lactate was identified as being more abundant in aerobic stationary phase *Z. mobilis *(Table 1, Fig. 2). Our data were also consistent with previous reports [28,29] showing metabolites such as lactate, acetate, and acetoin were more abundant during aerobic fermentation (Table 1, Fig. 2). Ishikawa et al. [19] proposed a model to explain the effect of oxygen on the NADH reducing power pool for the conversion of glucose into ethanol as the major end-product. They suggest that under aerobic conditions NADH is limiting due to it being oxidized by the NADH oxidase and therefore unavailable for reduction of acetaldehyde to ethanol. These data combined with lower intracellular glucose and ED pathway intermediates (Table 1) would agree with such a model and previous reports [30,31]. While we did not obtain the data for NAD^+^, NADH, ATP metabolites, altered redox balance with concomitant increases in other metabolites produced such as acetate, lactate, acetoin, and acetaldehyde in the presence of oxygen may account for lower ethanol production (Table 1, Fig. 2). We were able to observe differences in gene expression between exponential phase conditions by microarray, but not at levels considered significant using the Bonferroni multiple testing method (*p *< 0.05). Quantitative PCR assays showed greater sensitivity than array analyses but the majority of genes showed little difference in expression levels between aerobic and anaerobic conditions in early exponential phase. Each culture had more than doubled by the early exponential growth phase sampling point and this time point should have mitigated any potential inocula effects. The observation that there was an absence of a lag phase indirectly supports the idea that there was neither a large perturbation nor large regulatory response by the microbe upon inoculation into the fermentors (Fig. 1). The large differences in exponential and stationary phase transcriptomic profiles imply ZM4 is aerotolerant and oxygen affected the physiology of the cells leading to the buildup of metabolic byproducts, which ultimately led to greater expression differences in stationary phase.

Seo et al (2005) describe the *Z. mobilis *ZM4 (ATCC31821) genome as consisting of a single chromosome [16], which we utilized for probe design and microarray fabrication. The same *Z. mobilis *ZM4 (ATCC31821) strain utilized in this study contained plasmid DNA, which was in keeping with an earlier report describing the nomenclature and derivation of *Z. mobilis *strains [32]. Therefore, the array data in the present study may not fully represent the differences between aerobic and anaerobic conditions since these plasmid DNA sequences were unavailable. We used a multiplex array format with an average probe length of 36 nucleotides and were able to detect significantly differentially expressed genes. However, the use of shorter probes likely affected the sensitivity of the microarray analyses [33]. The proper identification of putative coding sequences and the use of systems biology tools will be important in identifying genome features that can be altered for improved ethanol production. The JGI's plans to sequence the genomes for additional *Zymomonas mobilis *strains, including strain ZM4 will likely improve gene prediction models and elucidate potential coding sequences present on plasmid DNA.

The effects of oxygen on *Z. mobilis *fermentation performance parameters and physiology have been examined in a number of previous studies [18-21,25,31,34-37]. However, this is the first report we are aware of that examines its effects on whole genome transcriptomic and metabolomic profiles of the bacterium under rigorously controlled conditions. The *Z. mobilis *strain CP4 *glf, zwf, edd *and *glk *genes for glucose uptake and utilization form an operon and coordinate gene expression appears to result from a complex pattern of mRNA degradation [38-40]. The ZM4 *glf, zwf, edd *and *glk *genes are similarly organized and all showed greater expression in stationary phase under anaerobic conditions, which was about 20, 9, 2, and 10-fold respectively more than that of aerobic condition, respectively. However *edd *differential expression did not meet our stringent significance criterion with a -log_10_(*p*) = 5.2. The *glf *gene encodes a glucose-facilitated diffusion transporter protein, which showed greater expression (approximately 20-fold) under anaerobic conditions even in the absence of detectable amounts of glucose in the medium at 26 h (Fig. 1). ZMO1859 showed 5-fold greater expression in the absence of oxygen. ZMO1859 is a putative OprB family carbohydrate-selective porin containing a pfam04966 (OprB family) conserved domain with an E-value score of 5e^-64 ^and 27% identity to *Pseudomonas aeruginosa *outer membrane protein OprB [41]. *P. aeruginosa *OprB transports glucose, mannitol, glycerol, and fructose. The *E. coli *expression of pathways for hexose uptake and metabolism has been shown to be up-regulated in the response to oxygen deprivation [30]. A number of ED and pyruvate biosynthetic pathway genes (*gntK, edd, eda, gap*) were showed higher expression levels under anaerobic conditions at 26 h when compared to the equivalent aerobic time point, but at levels not considered significant (see Additional file 3). Previous physiological studies have indicated that enzymatic activities for glucokinase, glucose-6-phosphate dehydrogenase, glyceraldehyde-3-phosphate dehydrogenase, ATPase, pyruvate kinase and alcohol dehydrogenase are decreased when oxygen partial pressure increases [31]. The intracellular metabolomic data suggests glucose present in anaerobic cells rapidly becomes phosphorylated, providing substrate for further glucose utilization (see Additional file 3). Expression of the *pdc *and *adhB *genes involved in *Z. mobilis *ethanol production was increased at least two-fold during anaerobic fermentation and was consistent with fermentation product and growth data (Fig. 1, 2, see Additional file 4).

These data suggest oxygen affects how carbon is utilized and partitioned in *Z. mobilis *under highly aerobic fermentations leading to the buildup of inhibitors such as acetaldehyde and acetate and to the accumulation of non-reducing sugars like trehalose that contribute to overall lower ethanol yields. The accumulation of acetate at the end of the aerobic fermentation was much more pronounced than seen in a number of previous studies, although the highly aerated fermentors with DOT measurement used in this study likely meant the culture conditions were quite different from previous batch fermentations without DOT measurement. In this study, we used an agitation rate of 700 rpm in conjunction with sparging air at 2.5 L/min to maintain fully aerobic conditions as previous experiments had demonstrated a lower agitation rate and this airflow rate was insufficient to maintain fully aerobic conditions as cell density increased (data not shown). Putative aldehyde dehydrogenase genes such as *ssdA *(ZMO1754) did not show differential expression in our study, but may potentially play a role in conversion of acetaldehyde to acetate in conjunction with possible reverse alcohol dehydrogenase reactions. However, much more detailed studies are required to understand the large accumulation of acetate under highly aerobic conditions.

Kalnenieks [11] reviewed the physiology of *Z. mobilis*, identified a number of unanswered questions about its energy metabolism, and determined that the structure and physiological role of the *Z. mobilis *respiratory chain remains to be fully elucidated. Kalnenieks et al [37] concluded that *Z. mobilis *contains a single NAD(P)H dehydrogenase encoded by *ndh *gene, and that its inhibition results in decreased respiration and improved aerobic growth. While we were not able to measure NAD, we did observe that *ndh *(ZMO1113) encoding NADH dehydrogenase was down-regulated in aerobic stationary phase cultures (see Additional file 4). A number of other respiratory genes were also down-regulated under these conditions. These included ZMO0022, ZMO1571, ZMO1572 and ZMO1844 encoding a putative Fe-S oxidoreductase, NADH dehydrogenase, cytochrome bd-type quinol oxidase subunits 1 and 2, and oxidoreductase genes, respectively (see Additional file 4). The stimulation of aerobic growth is proposed to be due to redirection of the NADH flux from respiration to ethanol synthesis resulting in less accumulation of toxic intermediates [11]. Several models have been proposed to account for the distribution of reducing equivalents between putative respiratory gene products and alcohol dehydrogenase reactions [11]. In one model both alcohol dehydrogenase isozymes catalyze ethanol synthesis and oxidation back to acetaldehyde and in another, the two isozymes operate in opposite directions. Under aerobic conditions, ethanol yield peaked around 13 h followed by net ethanol and acetaldehyde oxidation with concomitant increases in acetate and acetoin concentrations while approximately 0.66 mM of glucose remained in the medium at 26 h (Fig. 1, 2). We observed that expression of *adhB *(alcohol dehydrogenase II) in anaerobically cultured cells was approximately three-fold greater than when compared to aerobic cells at 26 h (see Additional file 4). *adhA *(alcohol dehydrogenase I) showed approximately 1.5-fold higher expression aerobically when compared to anaerobic cells, but did not meet our stringent criterion of a -log_10_(p-value) of 5.4. *Z. mobilis *contains two alcohol dehydrogenases, AdhB, an iron-containing alcohol dehydrogenase inactivated by oxygen and AdhA, a zinc-containing alcohol dehydrogenase that is resistant to oxidation [36]. Our *adhB *expression data were consistent with potential iron limitation and/or differential inactivation of AdhB under aerobic conditions.

We identified, for the first time to our knowledge, transcripts for the putative respiratory gene ZMO1814 (*rnfA*), encoding a putative NADH:ubiquinone oxidoreductase subunit that were expressed more greatly (3.3-fold) under aerobic conditions. The *Rhodobacter capsulatus rnf *gene products form a membrane complex involved in electron transfer to nitrogenase, however no physiological information is available for the *rnf *gene products of *Z. mobilis*, which does not fix nitrogen [42]. Although Sootsuwan et al. [42] demonstrate *Z. mobilis *has a cyanide-resistant terminal oxidase and could detect little difference between aerobic and anaerobic exponential phase ubiquinol oxidase activities, they documented significantly more ubiquinol oxidase activity in stationary phase cell membrane prepared anaerobically. We observed that expression of the *Z. mobilis *cytochrome bd-type quinol oxidase subunits genes (*cydA/B*) was slightly greater in stationary phase under anaerobic conditions. Trace amounts of oxygen, below detection limits, might have been present in anaerobic fermentors during stationary phase (see Additional file 1, 2). However, *E. coli cydAB *genes show greater expression under anaerobic conditions due to the change in negative supercoiling status and it has been suggested this may provide a mechanism for increasing cytochrome *bd *levels in response to environmental stress [43]. Sootsuwan et al. [42] proposed a number of possible physiological roles for a *Z. mobilis *respiratory chain including: controlling NADH levels to control ethanol production, reducing intracellular oxygen concentration and maintenance of NADH levels inside cells. The present study identified a number of putative genes involved in respiration as being expressed differentially depending upon conditions. However, it is clear much remains to be done to fully elucidate their function in redox balance and overall regulation. The contribution of individual genes in electron transfer and metabolic processes need to be followed up with targeted deletion mutant studies. The most surprising finding was the minimal impact of oxygen on the transcriptomic profiles in early exponential. Mutant studies would also be useful in assessing the physiology of early exponential growth phase aerobic *Z. mobilis *cells.

Ribitol was the most abundant differentially detected intracellular metabolite by GC-MS, at approximately 17-fold greater levels within aerobic cells compared to anaerobic cells (Table 1). The addition of various straight-chain alditols including ribitol has been reported to lead to the formation of novel *E. coli *phospholipids [44]. However, we did not conduct detailed phospholipid fatty acid (PLFA) analysis, nor target intact membranes. Ribitol and adenosine (latter was not considered significantly differentially expressed) are also both flavin adenine dinucleotide (FAD) components, which may reflect differences in redox potential and electron transfer between the two different conditions. Further studies are required to elucidate the role of ribitol and other metabolites like 4-hydroxybutanoate in *Z. mobilis *physiology and identify the unknown metabolites we detected. GC-MS based metabolomics provides a useful platform to examine a broad range of metabolites such as sugars, sugar alcohols, sugar acids, organic acids, amino acids, fatty acids, sterols, secondary metabolites, phenolic glycosides, alkaloids, purines, pyrimidines, and nucleosides [22,23,45,46]. However, a number of classes of metabolites cannot be detected by GC-MS and ribitol may not be the largest metabolite difference between these states. The differences of intracellular lactate indicated this metabolite was more abundant under aerobic conditions (Table 1), which was consistent with levels detected in the medium supernatant (Fig. 2).

Several ZM4 genes related to oxidative stress responses were up-regulated under aerobic, stationary phase conditions, including *ahpC *encoding alkyl hydroperoxide reductase, ZMO1097 encoding thioredoxin, and ZMO1118 encoding a glutathione S-transferase family protein (see Additional file 4). However, many systems involved in oxygen detoxification and redox homeostasis were not differentially expressed. There were no significant gene expression differences for classical oxidative stress response genes such as peroxidase (ZMO1573), catalase (ZMO0928), and iron-dependent superoxide dismutase (ZMO1060) when aerobic cultures were compared to anaerobic cultures. The glutathione system related genes such as *gor *(ZMO1211), *gshB *(ZMO1913) and ZMO1556 were also not affected significantly (data not shown). *Pyrococcus furiosus *exposed to gamma irradiation, which generates hydroxyl radicals and oxidative stress showed many systems involved in oxygen detoxification and redox homeostasis appeared to be constitutively expressed [47]. Furthermore, genes encoding superoxide dismutase, catalase, nonspecific peroxidases or thioredoxin reductase were not significantly expressed in response to air exposure in the obligate anaerobe *Methanosarcina barkeri *[48]. The *Z. mobilis *transcriptome analyses also suggested a relationship between aerobic conditions (or metabolic end-products) and cellular iron requirement (or limitation) as indicated by higher levels of expression of genes involved in iron sequestration and uptake. Transcripts for *Z. mobilis *iron-uptake and scavenging genes including *pbuA *(ZMO0188), *feoB *(ZMO1541), ZMO1463, and ZMO1847 (see Additional file 5) were all more abundant under aerobic conditions in stationary phase. A number of other studies such as those described by Brown et al [26] and Williams et al [47] have shown linkages between oxidative stress and expression of genes related to iron-uptake and storage.

The *Z. mobilis *ZMO1107 and ZMO0347 genes were up-regulated under anaerobic conditions (see Additional file 4) and are annotated as transcriptional regulator and hypothetical proteins, respectively [16]. BLASTP analyses indicated that ZMO1107 is similar to the *E. coli *global regulator leucine-responsive regulatory protein (Lrp) and ZMO0347 is similar to the *E. coli *global regulator Hfq, sharing about 40% and 60% identity to the *E. coli *Lrp and Hfq proteins, respectively. *E. coli *Lrp regulates transcription of many Lrp regulon genes with leucine as a co-regulator and also acts as a determinant of chromosome structure to coordinate cellular metabolism with the environmental nutritional state [49,50]. We observed different levels of intracellular leucine in the present study, but not at highly significant levels (Table 1). The best-studied Lrp family regulator is the *E. coli *Lrp, which affects the expression of at least 10% of all *E. coli *genes. Microarray analysis of gene expression profiles for *E. coli *Lrp^+ ^and Lrp^- ^strains indicates more than 400 genes are significantly Lrp-responsive with expression for 147 genes lower in Lrp^+ ^compared to Lrp^- ^cells [50,51]. In addition, Lrp has been suggested to play an important role in *E. coli *stationary phase regulation. Out of the 200 genes induced upon entrance into stationary phase, Lrp affects nearly three-quarters of them in *E. coli *[50]. It is well known that *E. coli *RpoS alternative sigma factor controls stationary phase gene expression [52,53]. The genome sequence shows that *Z. mobilis *has *rpoH *(ZMO0749), *rpoE *(ZMO1404), *rpoN *(ZMO0274), and *fliA *(ZMO0626, sigma F) genes but no identifiable *rpoS *gene [16]. ZMO1107 may play a role in stationary phase and stress regulation in *Z. mobilis*, but further studies are required to determine what functional overlap, if any, this protein has with RpoS-like functions in this bacterium.

A putative *Z. mobilis *Hfq global regulator was induced during anaerobic fermentation (Table 2). Hfq is a bacterial member of the Sm family of RNA-binding proteins, which acts by base-pairing with target mRNAs and functions as a chaperone for non-coding small RNA (sRNA) in *E. coli *[54-56]. Hfq is involved in regulating various processes and deletion of *hfq *has pleiotropic phenotypes, including slow growth, osmosensitivity, increased oxidation of carbon sources, and altered patterns of protein synthesis in *E. coli *[54,57]. *E. coli *Hfq has also been reported to affect genes involved in amino acid biosynthesis, sugar uptake, metabolism and energetics [58]. The expression of thirteen ribosomal genes was down-regulated in *hfq *mutant background in *E. coli *[58]. Hfq also up-regulated sugar uptake transporters and enzymes involved in glycolysis and fermentation such as *pgk *and *pykA*, and *adhE *[58]. Hfq is also involved in regulation of general stress responses that are mediated by alternative sigma factors such as RpoS, RpoE and RpoH. An *hfq *null mutant of *E. coli *exhibits strongly reduced RpoS levels by impairing *rpoS *translation [59]. Cells lacking Hfq induce the RpoE-mediated envelope stress response and *rpoH *is also induced in cells lacking Hfq in *E. coli *[58]. In our study, Hfq was less abundant in aerobic fermentation condition in ZM4 at 26 h post-inoculation (see Additional file 4) with eight ribosomal protein genes including *rplF, rplY*, and *rpmE *down-regulated (see Additional file 4). Meanwhile, glucose uptake genes such as *glf *and *oprB*, ED pathway genes such as *glk*, *zwf, pgl, eno*, and ethanol fermentation genes *pdc *and *adhB *were less abundant under aerobic condition in *Z. mobilis *(see Additional file 4). In addition, our microarray and qPCR data indicate that *rpoH *is also induced in aerobic condition (see Additional file 6) with low abundance of *hfq *transcripts (see Additional file 4). The association of Hfq with the expression of alternative sigma factor RpoH, ribosomal proteins and glycolytic enzyme in *Z. mobilis *is similar to that of *E. coli *as discussed above, which may indicate a similar conserved role of Hfq in both *E. coli *and *Z. mobilis*. However, further studies such as mutagenesis are required to elucidate the *Z. mobilis *Hfq regulon.

**Table 2:** Primer pairs used for q-PCR analysis with target gene information.

**Primers^1^**	**Sequences (5' to 3')**	**PCR product size (bp)**
ZMO0084_F	TGCAAGCATTGCCTACAAAG	100
ZMO0084_R	CCATTGAGGTGAACCCATCT	

ZMO0178_F	TGATCATCGGTGGTGGTATG	120
ZMO0178_R	TTTCAGCAGCAGCGAAAATA	

ZMO0367_F	ACAGCCTGATGAAACCATCC	111
ZMO0367_R	AACACATCCGTGAGGGAAAG	

ZMO0369_F	GCGTTTCTCTATTGCGGAAG	110
ZMO0369_R	CGAAACGTTCCCAAGCTAAC	

ZMO0749_F	TATCCTGCGGTCTTGGAGTC	108
ZMO0749_R	CCGTCTTCAAAGGCATTCAT	

ZMO0817_F	GCACCGAAATCAGCAAAAAT	107
ZMO0817_R	GTGTTGGGACTGGGTTCATC	

ZMO0976_F	CAGCAGATGGACGAGTTCAA	113
ZMO0976_R	TCGTTTTTCTTGGCATAGGG	

ZMO1062_F	AGGCCAATGACGGTTTACAG	115
ZMO1062_R	GCTTCCTGATCCAAAAGCTG	

ZMO1118_F	CGCCTGTTATTCTGGTGGAT	109
ZMO1118_R	CGCCTTATTTCAGCCCTATG	

ZMO1129_F	AGTGAAACCGACTGGCCTAA	104
ZMO1129_R	ATGGTTTCAATCGCAGCTCT	

ZMO1360_F	TGGTCTCAAGCATCACTTCG	114
ZMO1360_R	CCGCAGTTCAGTTCGTTACA	

ZMO1478_F	AGTAGCGGTGCTGAACTGGT	109
ZMO1478_R	CGGCAGGCCATTTATAAGAA	

ZMO1608_F	ACATGGCTAACGACGCTTCT	111
ZMO1608_R	TCTTGATCTGACCGCAGTTG	

ZMO1660_F	ATGAGCGTCCAGCAATTCTT	102
ZMO1660_R	TTCCCCGACATGACTCAGTA	

ZMO1863_F	GGAACTCTGGCAGAAACAGC	111
ZMO1863_R	GGATAACCCAAGACGAGCAA	

ZMO1876_F	ATCAGGATTTGACGCTGGAA	100
ZMO1876_R	ACCATCGCTTCGACAATAGC	

ZMO1959_F	ACAAGGCTGCCGATTTATTG	104
ZMO1959_R	TTGGCTCAGCAGATGTTGTC	

^1^PCR primer pair nomenclature. F: forward primer, R: reverse primer for each respective gene.

## Conclusion

Our study has provided insights into transcriptomic and metabolic profiles of the model ethanogenic bacterium *Z. mobilis *during aerobic and anaerobic fermentation under controlled fermentation conditions for the first time. It is unlikely the oxygen levels we used in the present study would be as high in an industrial setting, even in a major perturbation to the fermentation. However, our study showed oxygen affects maintenance energy, how carbon is utilized and partitioned in *Z. mobilis *fermentations and can lead to the buildup of inhibitors such as acetaldehyde and acetate under highly aerobic conditions, which contributed to lower ethanol yields. The most surprising finding was that while high oxygen concentrations resulted in slower growth, there seemed to be minimal impact on early exponential growth phase transcriptomic profiles. However, differences in energy-spilling pathways due to oxygen and uncoupled growth later led to large differences in ethanol yield and transcriptomic profiles between anaerobic and aerobic stationary phase cultures. Our study also identified a range of genes such as *cydA/B, rpoH *or ZMO0347 that could be targeted for deletion to better understand *Z. mobilis *physiology and coordinate regulation. Future studies examining the transcriptomic profiles for aerobic, microoxic and anaerobic steady states, the dynamics of transition between steady states and the use of mutant strains are warranted to provide greater insights into *Z. mobilis *physiology and gene regulation. Finally, our microarray based on the TIGR annotation has identified a number of genes that were not originally annotated as coding sequences in 2005 when the first *Z. mobilis *genome sequence was published [16], which included about 9% of the aerobic up-regulated genes (see Additional file 4, 5). The rapid accumulation of genome sequence information and sequencing technology advances make improving gene coding models and annotations even more important.

## Methods

### Bacterial strains and fermentation conditions

*Z. mobilis *ZM4 was obtained from the American Type Culture Collection (ATCC31821) and cultured in RM medium [60] at 30°C. For the inoculum preparation a single colony of ZM4 was added to a test tube containing 5 mL RM broth and cultured aerobically at 30°C until it reached late exponential or early stationary phase. A 1/100 dilution was added into the pre-warmed RM broth (10 mL culture into 1000 mL RM), which was then cultured aerobically at 30°C with shaking at 150 rpm for approximately 12 h. The optical density was measured with a spectrophotometer at 600_nm _and the inoculum was added to each fermentor so that the initial OD600_nm _was approximately 0.17 in each fermentor. Batch fermentations were conducted in approximately 2.5 L of RM medium in 7.5-L BioFlo110 bioreactors (New Brunswick Scientific, Edison, NJ) fitted with agitation, pH, temperature and DOT probes and controls. Culture pH was monitored using a pH electrode (Mettler-Toledo, Columbus, OH) and the pH control set point was maintained at 6.0 by automatic titration with 3 N KOH. Temperature was maintained automatically at 30°C and the vented gases exiting fermentors were passed through condenser units, chilled by a NESLAB Merlin M-150 refrigerated recirculator (Thermo Fisher Scientific, Newington, NH) to a vented hood via a water trap. DOT was monitored by using InPro 6800 series polarographic O_2 _sensors (Mettler-Toledo). Three anaerobic fermentors were sparged overnight with filter-sterilized N_2 _gas and for approximately one hour post-inoculation and the three aerobic fermentors were continually sparged with filter-sterilized air at 2.5 L/min to maintain fully aerobic conditions. The agitation rate was 700 rpm in each vessel.

### Growth, glucose and fermentation product analysis

Growth was monitored turbidometrically by measuring optical density at 600_nm _with a model 8453 spectrophotometer (Hewlett-Packard, Palo Alto, CA.). Fermentation media and fermentation products from filter-sterilized cell-free spent media were compositionally analyzed by either both the Amplex red glucose assay kit (Invitrogen, Carlsbad, CA) and high-performance liquid chromatography (HPLC) for glucose or by gas chromatography and/or HPLC for ethanol, acetate, lactate, acetaldehyde, and acetoin determinations. Glucose assays were incubated at room temperature for 30 minutes and performed according the manufacturer's instructions. Absorbance was detected using a microplate reader (model HTS 7000, PerkinElmer, Waltham, MA) at 590_nm_. Background absorbance was corrected by subtracting the value derived from the no-glucose control.

### Gas chromatography (GC)

Ethanol, acetate, acetaldehyde and acetoin concentrations in the medium supernatant were determined by flame ionization gas chromatography. Culture samples (1 mL) and standards were prepared by filtration and acidification with hydrochloric acid. The samples and standards were quantified by injecting 1 μL of each into a model 6890 Agilent Technologies equipped with a DB-FFAP 30 m × 0.53 mm × 1.5 μm film thickness capillary column (Agilent, Santa Clara, CA). The column operated with an initial temperature of 60°C and ramping 10°C to a final temperature of 180°C, while detector was at 250°C and injector temperature was 130°C with a post-injection dwell time of one minute. The carrier gas was helium at a constant flow rate of 5 mL/min.

### High-performance liquid chromatography (HPLC)

HPLC analysis was used for the measurements of the concentration of glucose, acetate, ethanol, and lactate in the 0.2 μm-filtered samples taken at different time points during fermentation. The diluted fermentation samples (1:1 with 8.98 mM sulfuric acid) were separated and quantified by HPLC using a LaChrom Elite System (Hitachi High Technologies America, Inc., San Jose, CA). Analysis was performed with an oven (Model L-2350) set at 60°C, and a pump (Model L-2130) set with a flow rate of 0.5 mL/min in 5 mM H_2_SO_4_. The run time for each sample was set for 35 minutes (Injector Model L-2200). Eluted compounds were registered and quantified by a refractive index detector (Model L-2490) equipped with a computer-powered integrator. Soluble fermentation products were identified by comparison with retention times and peak areas of corresponding standards. Metabolites were separated on an Aminex HPX-87H, 300 × 7.8 mm column (Bio-Rad, Hercules, CA).

### RNA isolation and preparation of fluorescein-labeled cDNA

RNA was isolated essentially described previously [26]. Briefly, samples from aerobic and anaerobic fermentors were harvested by centrifugation and the TRIzol reagent (Invitrogen, Carlsbad, CA) was used to extract total cellular RNA. Each total RNA preparation was treated with RNase-free DNase I (Ambion, Austin, TX) to digest residual chromosomal DNA and subsequently purified with the Qiagen RNeasy Mini kit in accordance with the instructions from the manufacturer. Total cellular RNA was quantified at OD_260 _and OD_280 _with a NanoDrop ND-1000 spectrophotometer (NanoDrop Technologies, Wilmington, DE). The purified RNA from each sample was used as the template to generate cDNA copies labeled with either Cy3-dUTP or Cy5-dUTP (GE Healthcare Bio-Sciences Corp, Piscataway, NJ). In a duplicate set of cDNA synthesis reactions the fluorescent dyes were reversed for each sample so that the effects of a specific dye were minimized. Labeling reaction components and incubation conditions as well as cDNA probe purification and concentration determination have been described previously [26].

### Microarray hybridization, scanning, image quantification, and data analyses

*Z. mobilis *microarrays were constructed by CombiMatrix Corporation (Mukilteo, WA) using coding sequences predicted by The Institute for Genomic Research (TIGR, ) as 101 more genes were predicted in this later genome annotation. Both the primary and TIGR annotations are presented in the present study. Microarray hybridizations were performed according to the manufacturer's instructions. Briefly, gene expression analysis was performed using six independent microarray experiments (two dye reversal reactions × three biological replicates) with each microarray containing one to two probes per predicted coding sequence each. The two separately labeled cDNA pools (i.e., the aerobic and the corresponding anaerobic time point) to be compared were mixed together in a hybridization solution containing 50% (v/v) formamide, applied to microarrays and incubated overnight (16 h) at 50°C. Microarrays were washed using buffers of increasing stringency according to the manufacturer's instructions, scanned with a ScanArray 5000 Microarray Analysis System (PerkinElmer Life Sciences Inc, Boston, MA) and the images were quantified using Microarray Imager software (CombiMatrix). Raw data was log_2 _transformed and imported into the statistical analysis software JMP Genomics 3.0 (SAS Institute, Cary, NC). A distribution analysis and data correlation analysis were done as a quality control step. The overlayed kernel density estimates derived from the distribution analysis allowed the visualization of sources of variation based on strain, as well as variation attributed to technical factors such as array and dye. The data were subsequently normalized using the standard normalization algorithm within JMP Genomics. An analysis of variance (ANOVA) was performed to determine differential expression levels between conditions and time points using the Bonferroni multiple testing method (*p *< 0.05).

### Quantitative-PCR (qPCR) analysis

Microarray data were validated using real-time qPCR as described previously [26]. Seventeen genes representing different functional categories and the range gene expression values, based on microarray hybridizations were analyzed using qPCR from cDNA derived from stationary phase samples. Primer pairs were designed as described previously, and the oligonucleotide sequences of the seventeen genes selected for qPCR analysis are listed in Table 2.

### Gas chromatography-mass spectrometry (GC-MS) metabolite analysis

Culture samples were rapidly pelleted by centrifugation, supernatants removed, cell pellets snap-frozen in liquid nitrogen and then stored at -80°C until analysis. Analyses were performed on microbial pellets collected in the stationary phase. A 5 mL aliquot of 80% ethanol (aqueous) was added to each pellet and cells disrupted using a sonicator 3000 (Misonix, Inc., Farmingdale, NY) operated at power level 5 for 6 times with a 1 min processing time and 1 min interval among each processing. An internal standard of 200 μL of sorbitol (1 mg/mL aqueous solution) was then added to each tube and 2 mL aliquots then dried in a helium stream. The internal standard was added to correct for differences in derivatization efficiency and changes in sample volume during heating. Dried exudates were dissolved in 500 μL of silylation-grade acetonitrile followed by the addition of 500 μL N-methyl-N-trimethylsilyltrifluoroacetamide (MSTFA) with 1% trimethylchlorosilane (TMCS) (Pierce Chemical Co., Rockford, IL), and samples then heated for 1 h at 70°C to generate trimethylsilyl (TMS) derivatives. After 1 day, 1-μL aliquots were injected into a ThermoFisher DSQII GC-MS, fitted with an Rtx-5MS (crosslinked 5% PH ME Siloxane) 30 m × 0.25 mm × 0.25 μm film thickness capillary column (Restek, Bellefonte, PA). The standard quadrupole GC-MS was operated in electron impact (70 eV) ionization mode, with 6 full-spectrum (70–650 Da) scans per second. Gas (helium) flow was set at 1.1 mL per minute with in injection port configured in the splitless mode. The injection port and detector temperatures were set to 220°C and 300°C, respectively. The initial oven temperature was held at 50°C for 2 min and was programmed to increase at 20°C per min to 325°C and held for another 11.25 min, before cycling back to the initial conditions. Quantified metabolites of interest were extracted using a key selected m/z that was characteristic for each metabolite, rather than the total ion chromatogram, to minimize integration of co-eluting metabolites. Peaks were quantified by area integration and the concentrations were normalized to the quantity of the internal standard (sorbitol) recovered, amount of sample extracted, derivitized, and injected. Two technical replicates were analyzed for two biological samples from each condition. Metabolite data of ZM4 under aerobic and anaerobic condition were averaged and presented as relative responses between ZM4 under aerobic fermentation versus ZM4 under anaerobic fermentation.

## Authors' contributions

SDB designed the microarray and conceived the experiment. SY and SDB performed the fermentation and sample collection. SY carried out the RNA extraction, microarray, qPCR, sample preparation for HPLC and GC-MS. MRJ performed HPLC analysis. TJT and NLE performed the GC-MS analysis. SLC performed GC-MS analysis. SLM assisted with microarray data analysis. SY and SDB analyzed the data and wrote the manuscript. BHD and AVP provided input on *Z. mobilis *metabolism and manuscript revision.

## Supplementary Material

Additional file 1 **Summary of fermentor parameters during 26 h fermentations.** Mean (± S.D.) agitation (rpm), temperature (°C), pH and dissolved oxygen tension values for three aerobic fermentors and three anaerobic fermentors averaged over the entire experiment. Click here for file

Additional file 2 **Dissolved oxygen tension during *Z. mobilis *fermentations.** Mean dissolved oxygen tension data for three aerobic fermentors and three anaerobic fermentors over 26 h. The bars represent the standard error of the mean data for each condition. Click here for file

Additional file 3 **Entner-Dondoroff and pyruvate metabolic pathways showing metabolomic and transcriptomic data at 26 h.** Summary of transcriptomic and metabolomic profiling data between aerobic and anaerobic conditions at 26 h. Click here for file

Additional file 4 **Aerobic down-regulated genes 26 h post inoculation.** Expression profiles for significantly differentially expressed genes that were down-regulated under aerobic conditions at 26 h as detected by microarrays and real-time qPCR. The modified gene function categories are based on MultiFun categories [Serres MH and Riley M: *Microb Comp Genomics *2000, **5**(4):18]. Click here for file

Additional file 5 **Aerobic up-regulated genes 26 h post inoculation.** Expression profiles for significantly differentially expressed genes that were up-regulated under aerobic conditions at 26 h as detected by microarrays and real-time qPCR. The modified gene function categories are based on MultiFun categories [Serres MH and Riley M: *Microb Comp Genomics *2000, **5**(4):18]. Click here for file

Additional file 6 **Comparison of exponential growth phase gene expression measurements by microarray and qPCR.** The gene expression ratios for wild-type *Z. mobilis *ZM4 under aerobic and anaerobic conditions after 3 h fermentation were log transformed in base 2 (log_2_<aerobic/anaerobic>). The microarray log_2 _ratio values (log_2_<aerobic/anaerobic>) were plotted against the qPCR log_2 _values. Comparison of the two methods indicated a level of concordance of R = 0.62. Click here for file

Additional file 7 **Comparison of stationary growth phase gene expression measurements by microarray and qPCR.** The gene expression ratios for wild-type *Z. mobilis *ZM4 under aerobic and anaerobic conditions after 26 h fermentation were log transformed in base 2 (log_2_<aerobic/anaerobic>). The microarray log_2 _ratio values (log_2_<aerobic/anaerobic>) were plotted against the qPCR log_2 _values. Comparison of the two methods indicated a high level of concordance (R = 0.92). Click here for file
