# Supplementary material for: Transcriptomic and metabolomic profiling of Zymomonas mobilis during aerobic and anaerobic fermentations
Source: BMC Genomics. 2009 Jan 20;10:34. doi: 10.1186/1471-2164-10-34 (PMC2651186; doi:10.1186/1471-2164-10-34)
Supplement: Additional file 1 — Summary of fermentor parameters during 26 h fermentations. Mean (± S.D.) agitation (rpm), temperature (°C), pH and dissolved oxygen tension values for three aerobic fermentors and three anaerobic fermentors averaged over the entire experiment. [file 1471-2164-10-34-S1.doc]

**Additional file 1**. Summary of fermentor parameters during 26h fermentations.

|  | **Aerobic**^1^ | **Anaerobic**^1^ |
| --- | --- | --- |
| **Agitation (rpm)** | 700 ± 0.03 | 700 ± 0.03 |
| **Temperature (°C)** | 30.0 ± 0.01 | 30.0 ± 0.03 |
| **pH** | 5.99 ± 0.04 | 6.04 ± 0.06 |
| **Dissolved O_2_ tension (DOT)** | 92.8 ± 5.9 | ND^2^ |

^1^Mean values ± standard deviation are reported for three independent fermentors for each condition.

^2^ DOT for the anaerobic fermentors was below detection of the polarographic O_2_ electrode. ND, not-detectable.
