# Supplementary figures and images for: Transcriptomic and metabolomic profiling of Zymomonas mobilis during aerobic and anaerobic fermentations
Source: BMC Genomics. 2009 Jan 20;10:34. doi: 10.1186/1471-2164-10-34 (PMC2651186; doi:10.1186/1471-2164-10-34)

## Slide 1
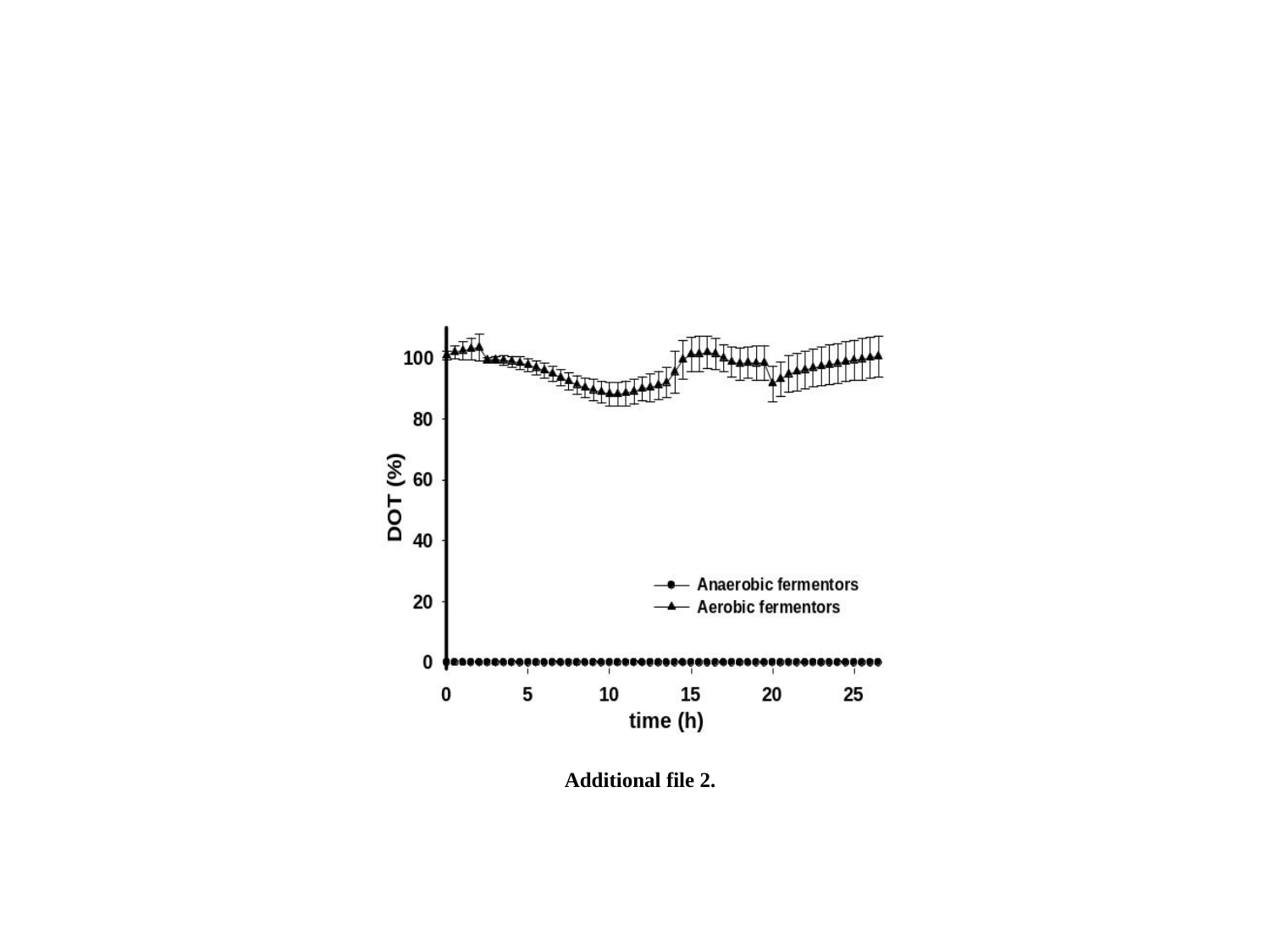

Additional file 2.

Supplement: Additional file 2 — Dissolved oxygen tension during Z. mobilis fermentations. Mean dissolved oxygen tension data for three aerobic fermentors and three anaerobic fermentors over 26 h. The bars represent the standard error of the mean data for each condition. [file 1471-2164-10-34-S2.ppt]

## Slide 1
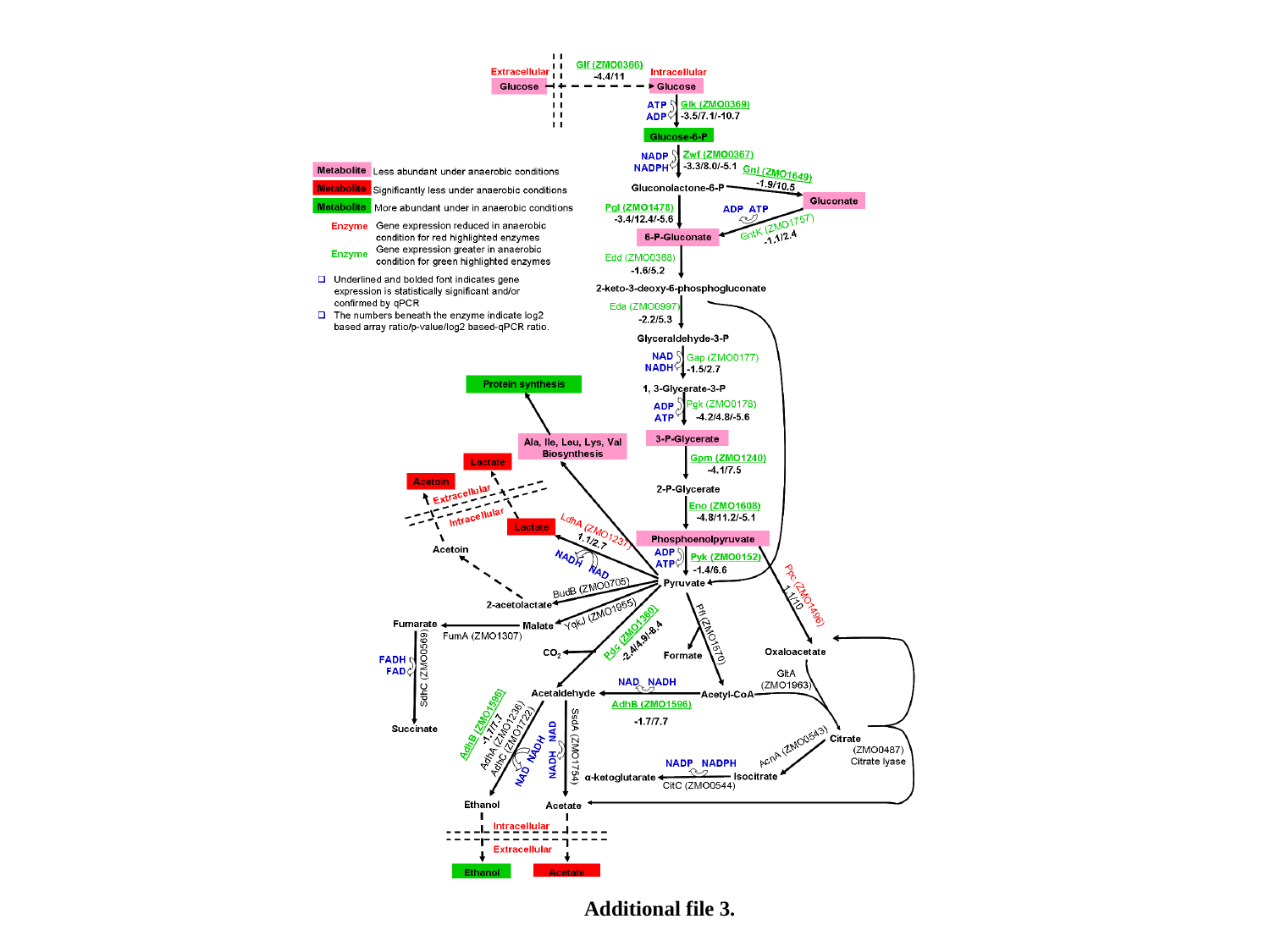

Additional file 3.

Supplement: Additional file 3 — Entner-Dondoroff and pyruvate metabolic pathways showing metabolomic and transcriptomic data at 26 h. Summary of transcriptomic and metabolomic profiling data between aerobic and anaerobic conditions at 26 h. [file 1471-2164-10-34-S3.ppt]

## Slide 1
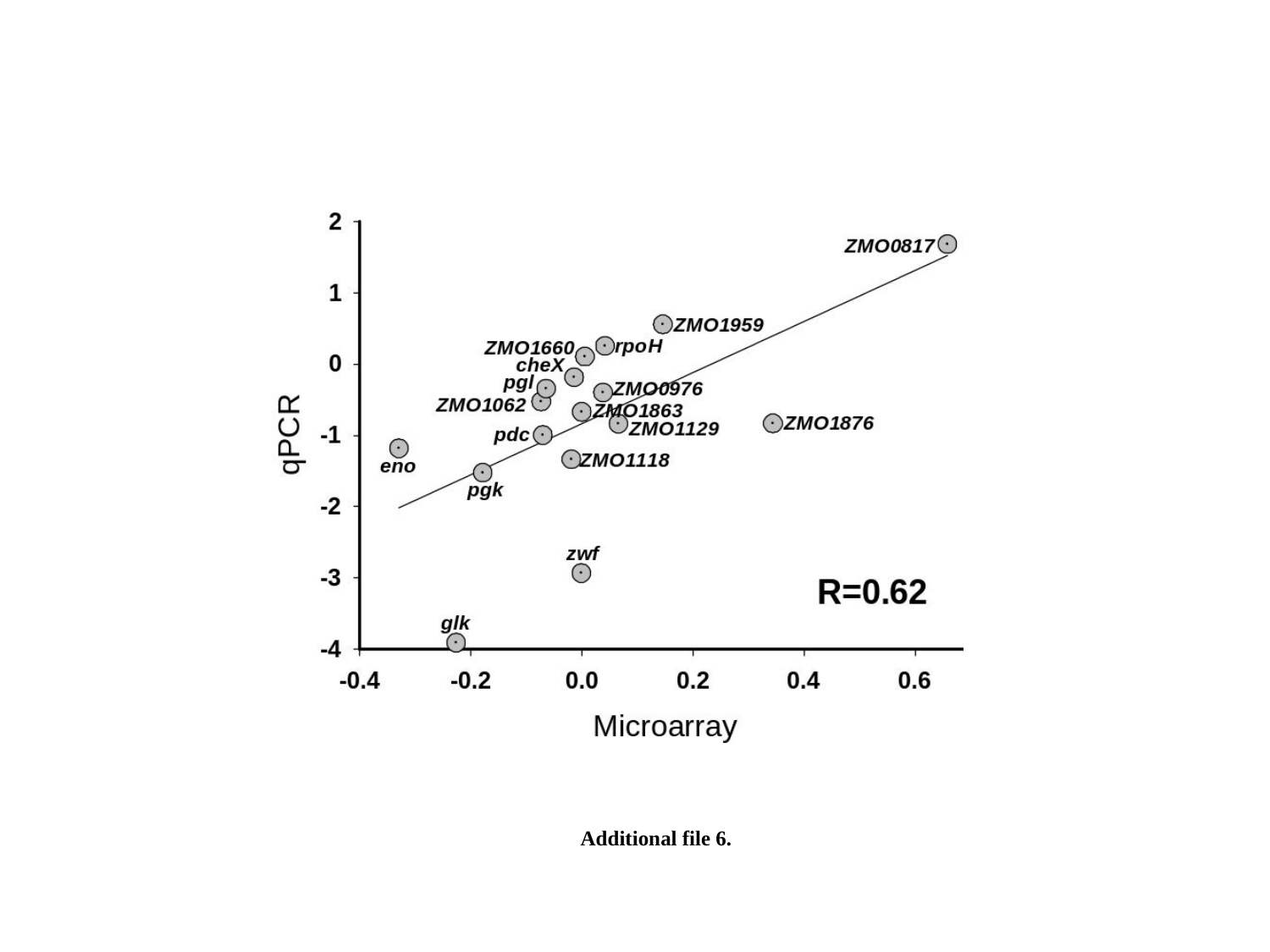

Additional file 6.

Supplement: Additional file 6 — Comparison of exponential growth phase gene expression measurements by microarray and qPCR. The gene expression ratios for wild-type Z. mobilis ZM4 under aerobic and anaerobic conditions after 3 h fermentation were log transformed in base 2 (log2<aerobic/anaerobic>). The microarray log2 ratio values (log2<aerobic/anaerobic>) were plotted against the qPCR log2 values. Comparison of the two methods indicated a level of concordance of R = 0.62. [file 1471-2164-10-34-S6.ppt]

## Slide 1
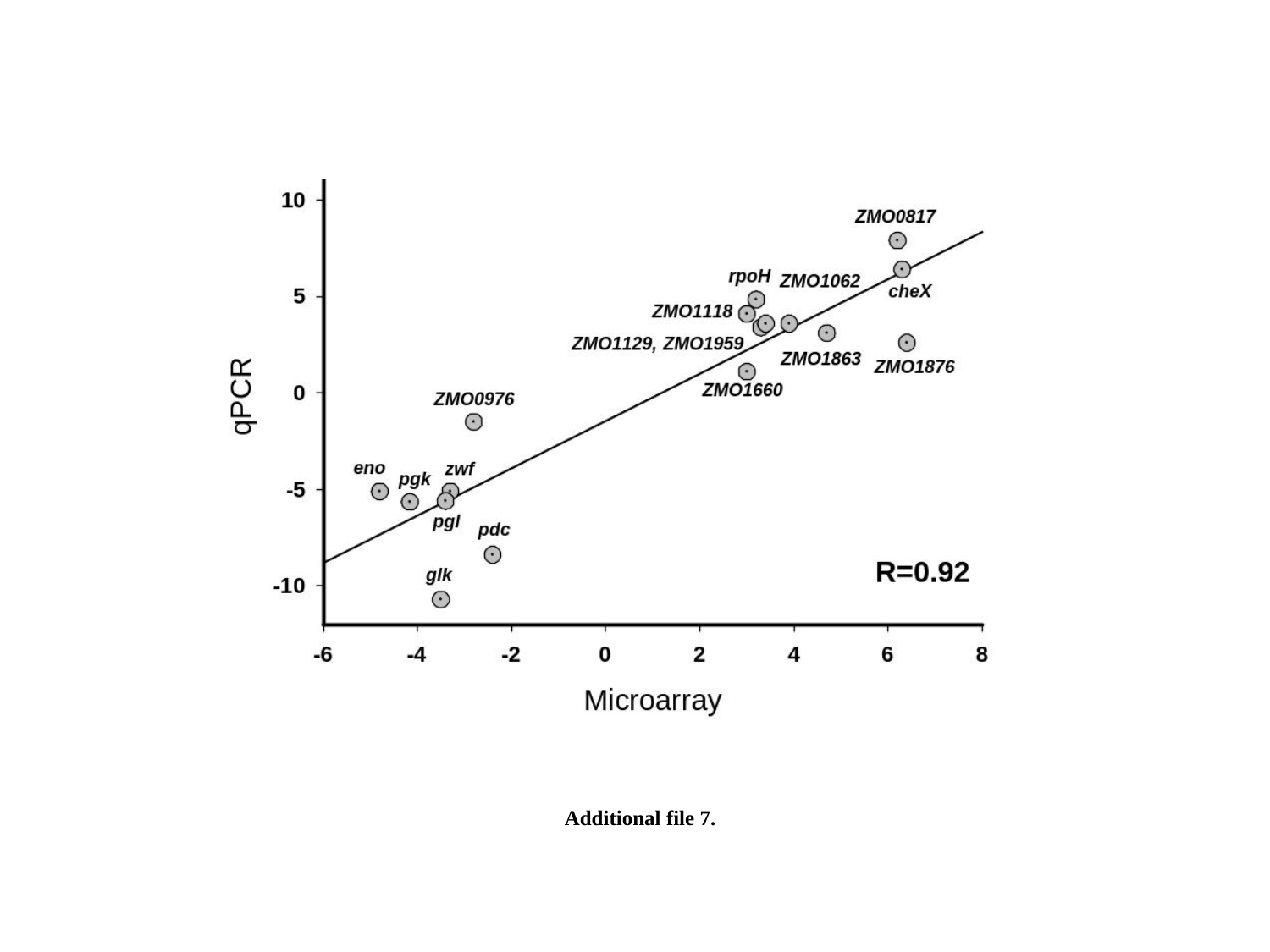

Additional file 7.

Supplement: Additional file 7 — Comparison of stationary growth phase gene expression measurements by microarray and qPCR. The gene expression ratios for wild-type Z. mobilis ZM4 under aerobic and anaerobic conditions after 26 h fermentation were log transformed in base 2 (log2<aerobic/anaerobic>). The microarray log2 ratio values (log2<aerobic/anaerobic>) were plotted against the qPCR log2 values. Comparison of the two methods indicated a high level of concordance (R = 0.92). [file 1471-2164-10-34-S7.ppt]
