# Supplementary material for: Transcriptomic and metabolomic profiling of Zymomonas mobilis during aerobic and anaerobic fermentations
Source: BMC Genomics. 2009 Jan 20;10:34. doi: 10.1186/1471-2164-10-34 (PMC2651186; doi:10.1186/1471-2164-10-34)
Supplement: Additional file 4 — Aerobic down-regulated genes 26 h post inoculation. Expression profiles for significantly differentially expressed genes that were down-regulated under aerobic conditions at 26 h as detected by microarrays and real-time qPCR. The modified gene function categories are based on MultiFun categories [Serres MH and Riley M: Microb Comp Genomics 2000, 5(4):18]. [file 1471-2164-10-34-S4.doc]

**Additional file 4**. Aerobic down-regulated genes 26 h post inoculation.

| **TIGR Locus** | **Primary Locus** | **Gene** | **Product** | **^a^EC#** | **^b^Array** | **^c^qPCR** |
| --- | --- | --- | --- | --- | --- | --- |
| **Metabolism** |  |  |  |  |  |  |
| NT01ZM0100 | ZMO0105 | *leuC* | 3-isopropylmalate dehydratase large subunit | 4.2.1.33 | -1.1 |  |
| NT01ZM0603 | ZMO0585 | *trpB* | Tryptophan synthase beta chain | 4.2.1.20 | -1.9 |  |
| NT01ZM0825 | ZMO0804 | *argC* | N-acetyl-gamma-glutamyl-phosphate reductase | 1.2.1.38 | -1.8 |  |
| NT01ZM1179 | ZMO1139 | *ilvI* | Acetolactate synthase large subunit | 2.2.1.6 | -1.3 |  |
| NT01ZM1181 | ZMO1141 | *ilvC* | Ketol-acid reductoisomerase | 1.1.1.86 | -6 |  |
| NT01ZM1464 | ZMO1407 | *asd* | Aspartate-semialdehyde dehydrogenase | 1.2.1.11 | -1.6 |  |
| NT01ZM1992 | ZMO1891 | *thrC* | Threonine synthase | 4.2.3.1 | -1 |  |
| NT01ZM0536 | ZMO0523 | *rplP* | Ribosomal protein L16/L10E |  | -3.8 |  |
| NT01ZM0541 | ZMO0528 | *rplE* | Ribosomal protein L5 |  | -4.4 |  |
| NT01ZM0544 | ZMO0531 | *rplF* | Ribosomal protein L6P/L9E |  | -4.9 |  |
| NT01ZM0552 | ZMO0539 | *rpsM* | Ribosomal protein S13 |  | -1.6 |  |
| NT01ZM0553 | ZMO0540 | *rpsK* | Ribosomal protein S11 |  | -3.9 |  |
| NT01ZM1185 | ZMO1145 | *rpmE* | Ribosomal protein L31 |  | -2.4 |  |
| NT01ZM1276 | ZMO1227 | *rplI* | Ribosomal protein L9 |  | -1.3 |  |
| NT01ZM2011 | ZMO1910 | *rplY* | Ribosomal protein L25 |  | -3.7 |  |
| NT01ZM0869 | NA |  | Arginyl-tRNA synthetase | 6.1.1.19 | -2.6 |  |
| NT01ZM1376 | ZMO1321 | *guaB* | Inosine-5-monophosphate dehydrogenase | 1.1.1.205 | -1.1 |  |
| NT01ZM0174 | ZMO0172 | *thiC* | Thiamine biosynthesis protein |  | -1.7 |  |
| NT01ZM0482 | ZMO0475 | *ribC* | Riboflavin synthase alpha chain | 2.5.1.9 | -1.5 |  |
| NT01ZM0915 | ZMO0889 | *mro* | Aldose 1-epimerase precursor | 5.1.3.3 | -1.4 |  |
| NT01ZM0247 | ZMO0239 | *atpA* | ATP synthase alpha subunit | 3.6.3.14 | -1.7 |  |
| NT01ZM0249 | ZMO0241 | *atpD* | ATP synthase beta subunit | 3.6.3.14 | -1.8 |  |
| NT01ZM1633 | ZMO1571 | *cydA* | Cytochrome bd-type quinol oxidase subunit 1 | 1.10.3.- | -1.6 |  |
| NT01ZM1634 | ZMO1572 | *cydB* | Cytochrome bd-type quinol oxidase subunit 2 | 1.10.3.- | -1.5 |  |
| NT01ZM1151 | ZMO1113 | *ndh* | NADH dehydrogenase | 1.6.99.3 | -2 |  |
| NT01ZM0147 | ZMO0152 | *pyk* | Pyruvate kinase | 2.7.1.40 | -1.4 |  |
| NT01ZM0371 | ZMO0367 | *zwf* | Glucose-6-phosphate dehydrogenase | 1.1.1.49 | -3.3 | -5.1 |
| NT01ZM0373 | ZMO0369 | *glk* | Glucokinase | 2.7.1.2 | -3.5 | -10.7 |
| NT01ZM0473 | ZMO0465 | *tpi* | Triosephosphate isomerase | 5.3.1.1 | -1.4 |  |
| NT01ZM1290 | ZMO1240 | *gpm* | Phosphoglycerate mutase | 5.4.2.1 | -4.1 |  |
| NT01ZM1537 | ZMO1478 | *pgl* | 6-phosphogluconolactonase | 3.1.1.31 | -3.4 | -5.6 |
| NT01ZM1665 | ZMO1596 | *adhB* | Alcohol dehydrogenase II | 1.1.1.1 | -1.7 |  |
| NT01ZM1677 | ZMO1608 | *eno* | Enolase | 4.2.1.11 | -4.8 | -5.1 |
| NT01ZM1725 | ZMO1649 | *gnl* | Gluconolactonase | 3.1.1.17 | -1.9 |  |
| NT01ZM1798 | ZMO1719 | *frk* | Fructokinase | 2.7.1.4 | -3.8 |  |
| **Cell Process** |  |  |  |  |  |  |
| NT01ZM0711 | ZMO0693 |  | Organic hydroperoxide resistance protein |  | -6.8 |  |
| NT01ZM0629 | ZMO0611 | *flgE* | Flagellar hook protein |  | -2.1 |  |
| NT01ZM1653 | ZMO1586 | *bfr* | Bacterioferritin |  | -2.1 |  |
| **Information transfer** | |  |  |  |  |  |
| NT01ZM0353 | ZMO0347 |  | RNA-binding protein Hfq |  | -1.1 |  |
| NT01ZM0753 | ZMO0732 |  | DNA-directed RNA polymerase beta subunit | 2.7.7.6 | -2.1 |  |
| NT01ZM1145 | ZMO1107 |  | Transcriptional regulator |  | -1.6 |  |
| **Transporter** |  |  |  |  |  |  |
| NT01ZM0370 | ZMO0366 | *glf* | Glucose facilitated diffusion protein |  | -4.4 |  |
| NT01ZM0164 | ZMO0165 | *tolB* | Tol biopolymer transport system |  | -2.3 |  |
| NT01ZM1951 | ZMO1859 | *rpfN* | Carbohydrate-selective porin |  | -2.6 |  |
| **Unknown** |  |  |  |  |  |  |
| NT01ZM1011 | ZMO0976 |  | Putative oxidoreductase |  | -2.8 | -1.5 |
| NT01ZM0324 | ZMO0318 |  | Oxidoreductase | 1.1.1.100 | -3.3 |  |
| NT01ZM0901 | ZMO0874 |  | Predicted Fe-S oxidoreductase |  | -1.2 |  |
| NT01ZM0022 | ZMO0022 |  | Fe-S oxidoreductase |  | -2.1 |  |
| NT01ZM1931 | ZMO1844 |  | Probable oxidoreductase |  | -1.9 |  |
| NT01ZM1863 | ZMO1779 |  | Hypothetical protein |  | -6.3 |  |
| NT01ZM1649 | ZMO1581 |  | Hypothetical protein |  | -2.2 |  |
| NT01ZM0687 | ZMO0670 |  | Hypothetical protein |  | -3.6 |  |
| NT01ZM1678 | ZMO1609 |  | Hypothetical protein |  | -2.9 |  |
| NT01ZM1148 | ZMO1109 |  | Hypothetical protein |  | -2.5 |  |
| NT01ZM1359 | ZMO1305 |  | Hypothetical protein |  | -2.1 |  |

^a^EC#: Enzyme commission number;  ^b^Array: the log_2_ based microarray ratio of the gene expression (aerobic/anaerobic);  ^c^qPCR: the log_2_ based qPCR ratio of the gene expression (aerobic/anaerobic); NA: not-annotated.
