# Supplementary material for: Transcriptomic and metabolomic profiling of Zymomonas mobilis during aerobic and anaerobic fermentations
Source: BMC Genomics. 2009 Jan 20;10:34. doi: 10.1186/1471-2164-10-34 (PMC2651186; doi:10.1186/1471-2164-10-34)
Supplement: Additional file 5 — Aerobic up-regulated genes 26 h post inoculation. Expression profiles for significantly differentially expressed genes that were up-regulated under aerobic conditions at 26 h as detected by microarrays and real-time qPCR. The modified gene function categories are based on MultiFun categories [Serres MH and Riley M: Microb Comp Genomics 2000, 5(4):18]. [file 1471-2164-10-34-S5.doc]

**Additional file 5**. Aerobic up-regulated genes 26 h post inoculation.

| **TIGR Locus** | **Primary Locus** | **Gene** | **Product** | **^a^EC#** | **^b^Array** | **^c^qPCR** |
| --- | --- | --- | --- | --- | --- | --- |
| **Metabolism** |  |  |  |  |  |  |
| NT01ZM1939 | ZMO1853 | *dapA* | Dihydrodipicolinate synthase | 4.2.1.52 | 1.4 |  |
| NT01ZM0318 | ZMO0311 | *proC* | Pyrroline-5-carboxylate reductase | 1.5.1.2 | 2 |  |
| NT01ZM1877 | ZMO1792 | *ilvD* | Dihydroxy-acid dehydratase | 4.2.1.9 | 2.4 |  |
| NT01ZM0257 | ZMO0251 |  | Putative translation initiation inhibitor |  | 1.6 |  |
| NT01ZM0769 | ZMO0746 |  | Aspartyl-tRNA synthetase |  | 1.4 |  |
| NT01ZM1688 | ZMO1617 | *carB* | Carbamoyl-phosphate synthase large chain | 6.3.5.5 | 1.6 |  |
| NT01ZM1412 | ZMO1358 |  | Ribosomal protein S20 |  | 2 |  |
| NT01ZM0216 | ZMO0209 | *rpmA* | Ribosomal protein L27 |  | 2.4 |  |
| NT01ZM0255 | ZMO0249 |  | Ribosomal protein L33 |  | 4 |  |
| NT01ZM1987 | ZMO1887 | *entB* | Isochorismatase | 3.3.2.1 | 1.1 |  |
| NT01ZM1060 | ZMO1022 | *nifS* | Cysteine desulfurase | 4.4.1.2 | 1.7 |  |
| NT01ZM0926 | ZMO0899 |  | NAD+ synthetase | 6.3.1.5 | 3.3 |  |
| NT01ZM1978 | ZMO1879 | *hemB* | Delta-aminolevulinic acid dehydratase | 4.2.1.24 | 5.1 |  |
| NT01ZM1953 | ZMO1861 |  | Enoyl-(acyl-carrier-protein) reductase II |  | 3.1 |  |
| NT01ZM1548 | ZMO1489 | *kdsB* | 3-deoxy-D-manno-octulosonate cytidylyltransferase | 2.7.7.38 | 2.5 |  |
| NT01ZM1127 | ZMO1088 |  | Putative rare lipoprotein A |  | 2.5 |  |
| NT01ZM0777 | ZMO0754 |  | SCP-2 sterol transfer family superfamily |  | 3.1 |  |
| NT01ZM1282 | ZMO1232 |  | Glycosyltransferase |  | 1.7 |  |
| NT01ZM1521 | ZMO1460 | *sseA* | Thiosulfate sulfurtransferase | 2.8.1.1 | 1.3 |  |
| NT01ZM1955 | ZMO1863 |  | Putative phosphatase | 3.1.3.18 | 4.7 | 3.1 |
| NT01ZM1556 | ZMO1496 | *ppc* | Phosphoenolpyruvate carboxylase | 4.1.1.31 | 1.1 |  |
| NT01ZM1401 | ZMO1347 | *ltaE* | Threonine aldolase | 4.1.2.5 | 2.2 |  |
| NT01ZM1938 | ZMO1851 | *nifF* | Flavodoxin |  | 2.3 |  |
| NT01ZM1900 | ZMO1814 | *rnfA* | NADH:ubiquinone oxidoreductase subunit |  | 3.3 |  |
| NT01ZM1638 | ZMO1576 |  | Short-chain dehydrogenase | 1.-.-.- | 2.2 |  |
| NT01ZM1467 | NA |  | BFD-like [2Fe-2S] binding domain superfamily |  | 2 |  |
| NT01ZM1337 | ZMO1286 | *sldS* | Sorbitol dehydrogenase small subunit | 1.1.99.21 | 2.3 |  |
| **Cell Process** |  |  |  |  |  |  |
| NT01ZM1765 | ZMO1688 |  | Glycine cleavage T protein | 2.1.2.10 | 1.7 |  |
| NT01ZM1338 | ZMO1287 |  | LPS glycosyltransferase | 2.4.1.- | 4.4 |  |
| NT01ZM1128 | ZMO1089 | *dacC* | D-alanyl-D-alanine carboxypeptidase | 3.4.16.4 | 1.5 |  |
| NT01ZM2005 | ZMO1904 |  | Metal-dependent protease |  | 1.2 |  |
| NT01ZM1136 | ZMO1097 |  | Thioredoxin |  | 1.9 |  |
| NT01ZM1157 | ZMO1118 |  | Glutathione S-transferase family protein | 2.5.1.18 | 3 | 4.1 |
| NT01ZM0439 | ZMO0433 | *gmk* | Guanylate kinase | 2.7.4.8 | 1.6 |  |
| NT01ZM1810 | ZMO1732 | *ahpC* | Alkyl hydroperoxide reductase |  | 4.2 |  |
| NT01ZM0288 | ZMO0279 |  | Cold shock protein |  | 1.2 |  |
| NT01ZM0080 | ZMO0084 |  | CheX protein |  | 6.3 | 6.4 |
| NT01ZM0695 | ZMO0678 | *tdsD* | Nitroreductase |  | 1.4 |  |
| NT01ZM0659 | ZMO0641 | *motD* | Chemotaxis protein |  | 1.7 |  |
| NT01ZM0668 | ZMO0651 | *fliD* | Flagellar hook-associated protein 2 |  | 3.2 |  |
| NT01ZM0997 | ZMO0964 |  | Probable multidrug resistance lipoprotein |  | 1.6 |  |
| **Information transfer** | |  |  |  |  |  |
| NT01ZM1160 | ZMO1121 |  | Bacterial regulatory protein, MerR family |  | 1.3 |  |
| NT01ZM1264 | ZMO1216 |  | Two-component signal transduction histidine kinase |  | 2.2 |  |
| NT01ZM1442 | ZMO1387 |  | Two-component response regulator |  | 2.2 |  |
| NT01ZM1799 | ZMO1720 | *rpoZ* | DNA-directed RNA polymerase omega subunit | 2.7.7.6 | 2.4 |  |
| NT01ZM1102 | ZMO1063 | *pspA* | Sigma 54-dependent transcription suppressor |  | 2.4 |  |
| NT01ZM0647 | ZMO0630 |  | Torf protein |  | 4.7 |  |
| NT01ZM1694 | ZMO1622 | *dnaG* | DNA primase | 2.7.7.- | 1.9 |  |
| **Transporter** |  |  |  |  |  |  |
| NT01ZM1088 | ZMO1048 | *pstc* | Phosphate ABC transporter permease |  | 2.2 |  |
| NT01ZM1524 | ZMO1463 |  | Probable TonB-dependent receptor |  | 1.1 |  |
| NT01ZM1600 | ZMO1541 | *feoB* | Ferrous iron transport protein B |  | 3.8 |  |
| NT01ZM1934 | ZMO1847 |  | ABC Fe3+ transport system permease |  | 1.8 |  |
| NT01ZM0193 | ZMO0188 | *pbuA* | Ferric-pseudobactin M114 receptor precursor |  | 2.6 |  |
| NT01ZM1942 | ZMO1856 |  | MFS subfamily transporter |  | 5.1 |  |
| NT01ZM1488 | ZMO1430 |  | Multidrug resistance efflux pump |  | 1.2 |  |
| NT01ZM0295 | ZMO0285 |  | RND efflux system lipoprotein |  | 2 |  |
| NT01ZM1496 | ZMO1437 |  | LysE family transporter |  | 1.6 |  |
| NT01ZM1982 | ZMO1882 |  | Putative transport protein |  | 3.1 |  |
| **Unknown** |  |  |  |  |  |  |
| NT01ZM1952 | ZMO1860 |  | Nodulin 21 like protein |  | 2 |  |
| NT01ZM0786 | ZMO0763 |  | TPR Domain domain protein |  | 2 |  |
| NT01ZM0325 | ZMO0319 |  | WGR domain superfamily |  | 3.1 |  |
| NT01ZM1917 | ZMO1830 | *fdxB* | Ferredoxin |  | 2.5 |  |
| NT01ZM0810 | ZMO0786 |  | Dehydrogenase subunit III, putative |  | 1.3 |  |
| NT01ZM1212 | ZMO1170 |  | Vng6254c |  | 2.8 |  |
| NT01ZM0424 | ZMO0418 |  | Uncharacterized ACR, COG1434 family |  | 1.2 |  |
| NT01ZM1043 | ZMO1007 |  | Uncharacterized protein family (UPF0187) |  | 1.5 |  |
| NT01ZM2064 | ZMO1959 |  | ATPase |  | 3.4 | 3.6 |
| NT01ZM0838 | ZMO0817 |  | Hypothetical protein |  | 6.2 | 7.9 |
| NT01ZM1169 | ZMO1129 |  | Hypothetical protein |  | 3.3 | 3.4 |
| NT01ZM1974 | ZMO1876 |  | Hypothetical protein |  | 6.4 | 2.6 |
| NT01ZM1736 | ZMO1660 |  | Hypothetical protein |  | 3 | 1.1 |
| NT01ZM1101 | ZMO1062 |  | Hypothetical protein |  | 3.9 | 3.6 |
| NT01ZM0463 | NA |  | Hypothetical protein |  | 1.1 |  |
| NT01ZM1973 | NA |  | Hypothetical protein |  | 1.8 |  |
| NT01ZM0274 | NA |  | Hypothetical protein |  | 1.8 |  |
| NT01ZM0922 | NA |  | Hypothetical protein |  | 2.1 |  |
| NT01ZM0935 | NA |  | Hypothetical protein |  | 2.2 |  |
| NT01ZM1663 | NA |  | Hypothetical protein |  | 2.4 |  |
| NT01ZM0198 | NA |  | Hypothetical protein |  | 2.5 |  |
| NT01ZM0523 | NA |  | Hypothetical protein |  | 4.4 |  |
| NT01ZM1532 | NA |  | Hypothetical protein |  | 4.4 |  |
| NT01ZM0573 | ZMO0557 |  | Hypothetical protein |  | 2.3 |  |
| NT01ZM1937 | ZMO1850 |  | Hypothetical protein |  | 1.8 |  |
| NT01ZM0701 | ZMO0683 |  | Hypothetical protein |  | 1.2 |  |
| NT01ZM0698 | ZMO0681 |  | Hypothetical protein |  | 1.5 |  |
| NT01ZM1198 | ZMO1157 |  | Hypothetical protein |  | 1.9 |  |
| NT01ZM0358 | ZMO0352 |  | Hypothetical protein |  | 2.9 |  |
| NT01ZM1119 | ZMO1080 |  | Hypothetical protein |  | 1.0 |  |
| NT01ZM1263 | ZMO1215 |  | Hypothetical protein |  | 1.1 |  |
| NT01ZM0107 | ZMO0112 |  | Hypothetical protein |  | 1.2 |  |
| NT01ZM1566 | ZMO1506 |  | Hypothetical protein |  | 1.2 |  |
| NT01ZM1626 | ZMO1565 |  | Hypothetical protein |  | 1.2 |  |
| NT01ZM0411 | ZMO0404 |  | Hypothetical protein |  | 1.5 |  |
| NT01ZM1387 | ZMO1332 |  | Hypothetical protein |  | 1.5 |  |
| NT01ZM1747 | ZMO1671 |  | Hypothetical protein |  | 1.6 |  |
| NT01ZM1031 | ZMO0995 |  | Hypothetical protein |  | 1.6 |  |
| NT01ZM2002 | ZMO1901 |  | Hypothetical protein |  | 1.7 |  |
| NT01ZM0034 | ZMO0034 |  | Hypothetical protein |  | 1.7 |  |
| NT01ZM1881 | ZMO1795 |  | Hypothetical protein |  | 1.8 |  |
| NT01ZM1994 | ZMO1894 |  | Hypothetical protein |  | 1.8 |  |
| NT01ZM1142 | ZMO1104 |  | Hypothetical protein |  | 1.9 |  |
| NT01ZM0639 | ZMO0621 |  | Hypothetical protein |  | 2.0 |  |
| NT01ZM1981 | ZMO1881 |  | Hypothetical protein |  | 2.1 |  |
| NT01ZM1664 | ZMO1595 |  | Hypothetical protein |  | 2.2 |  |
| NT01ZM1857 | ZMO1774 |  | Hypothetical protein |  | 2.2 |  |
| NT01ZM1666 | ZMO1597 |  | Hypothetical protein |  | 2.4 |  |
| NT01ZM1693 | ZMO1621 |  | Hypothetical protein |  | 2.9 |  |
| NT01ZM1251 | ZMO1204 |  | Hypothetical protein |  | 3.0 |  |
| NT01ZM1525 | ZMO1464 |  | Hypothetical protein |  | 3.1 |  |
| NT01ZM1202 | ZMO1161 |  | Hypothetical protein |  | 3.1 |  |
| NT01ZM0836 | ZMO0815 |  | Hypothetical protein |  | 3.2 |  |

^a^EC#: Enzyme commission number;  ^b^Array: the log_2_  based microarray ratio of the gene expression (aerobic/anaerobic);  ^c^qPCR: the log_2_ based qPCR ratio of the gene expression (aerobic/anaerobic); NA: not-annotated.
